# Comparing the expression profiles of steroid hormone receptors and stromal cell markers in prostate cancer at different Gleason scores

**DOI:** 10.1038/s41598-018-32711-9

**Published:** 2018-09-25

**Authors:** Thomas Gevaert, Yves-Rémi Van Eycke, Thomas Vanden Broeck, Hein Van Poppel, Isabelle Salmon, Sandrine Rorive, Frank Claessens, Dirk De Ridder, Christine Decaestecker, Steven Joniau

**Affiliations:** 1 Department of Urology, UZ Leuven, Leuven, Belgium; 2 Organ Systems, KU Leuven, Leuven, Belgium; 3 Department of Pathology, AZ Klina, Brasschaat, Belgium; 4 P.E.A.R.L. (ProstatE cAncer Research Leuven), UZ Leuven, Leuven, Belgium; 5 Laboratories of Image, Signal processing and Acoustics (LISA), Brussels School of Engineering/École polytechnique de Bruxelles, ULB, Brussels, Belgium; 6 DIAPath - Center for Microscopy and Molecular Imaging (CMMI), ULB, Gosselies, Belgium; 7 Department of Pathology, Erasme University Hospital, Université Libre de Bruxelles (ULB), Brussels, Belgium; 8 Centre Universitaire Inter Régional d’Expertise en Anatomie Pathologique Hospitalière (CurePath), Jumet, Belgium; 9 Department of Molecular and Cellular Medicine, KU Leuven, Leuven, Belgium

## Abstract

The recent developments in anti-angiogenic and immunomodulatory drugs show that the tumour micro-environment (TME) becomes increasingly important in cancer research. Here we investigated the correlation between the Gleason score (GS) and the TME by comparing tissue expression profiles of steroid hormone receptors, cancer activated fibroblast (CAF) markers and vessel densities between different GS groups. Therefore, matched patient cohorts were composed for different GS (6-7-8). Tissue micro-arrays with 6 samples/patient were processed for immunohistochemistry. Stained slides were digitised, stroma and epithelium were selectively annotated, and all selected areas were quantitatively analysed for marker expression. The most striking findings were decreased stromal expression levels of several steroid hormone receptors, increased CAF-phenotypes and increased vessel densities in high GS prostate cancer compared to low GS prostate cancer and paired prostate non-tumour tissue. The present data reveal a complex correlation between prostate cancer differentiation and TME components and suggest that different GS can be associated with different possible actionable targets in the TME. The use of standardised digital image analysis tools generated robust and reproducible quantitative data, which is novel and more informative compared to the classic semi-quantitative and observer-dependent visual scoring of immunohistochemistry.

## Introduction

The Gleason score (GS) system and the Grade Group system recently introduced by the international society for uropathology (ISUP) are still the mainstay of prostate cancer (PCa) grading^1,2^. The ISUP Grade Group system recognizes five distinct grade groups based on the classic GS system and has the advantage to offer a simplified and more straightforward classification^1^. When added to clinical stage and serum PSA level, the Gleason grading remains a powerful prognostic marker to guide therapy decision for PCa^3^.

The key element in the GS is the evaluation of the morphology of tumour glands. It is intriguing that this longstanding methodology remains such a powerful prognostic tool. During the past years our knowledge of the molecular features of PCa and of the role of the tumour micro-environment (TME) in PCa progression has gradually expanded^4,5^. This TME consists of an interconnected network of stromal fibroblasts, immune cells, blood vessels, mesenchymal stem cells (MSCs), pericytes, fat cells, neural cells and secreted soluble and insoluble factors such as chemokines, cytokines and extracellular matrices^5,6^. Interactions between neoplastic cells and the TME are complex and change progressively during the multistep transformation of normal cells into high-grade malignancies and the subsequent cancer dissemination process^6^.

Many studies revealed relations between GS and the TME, including GS-dependent changes in expression of steroid hormone receptors (SHR)^7–10^, cancer activated fibroblast (CAF) markers^11–13^ and vascular markers^13,14^. However, most of these studies focus on individual markers and/or pathways, and therefore transversal studies crossing the relation between GS and the different key elements of the TME are lacking. In the present study we investigated the relation between GS and established TME markers by comparing tissue expression profiles of steroid hormone receptors (SHR: androgen receptor (AR), progesterone receptor (PR) and estrogen receptor alpha (ERα)), CAF markers (CD34, caveolin-1 (CAV-1) and alpha smooth muscle actin (αSMA)) and the vascular marker CD31 in paired PCa and prostate non-tumour (PNT) tissue. Immunohistochemistry (IHC) is an important part of the methodology to study the TME. We aimed to generate robust quantitative IHC data using calibrated image acquisition and validated image analysis algorithms, as reported previously^15^.

## Results

In all the results below protein expression is quantified in terms of the labelling index (LI) which is representative of the percentage of positive cells.

**Figure 1:**
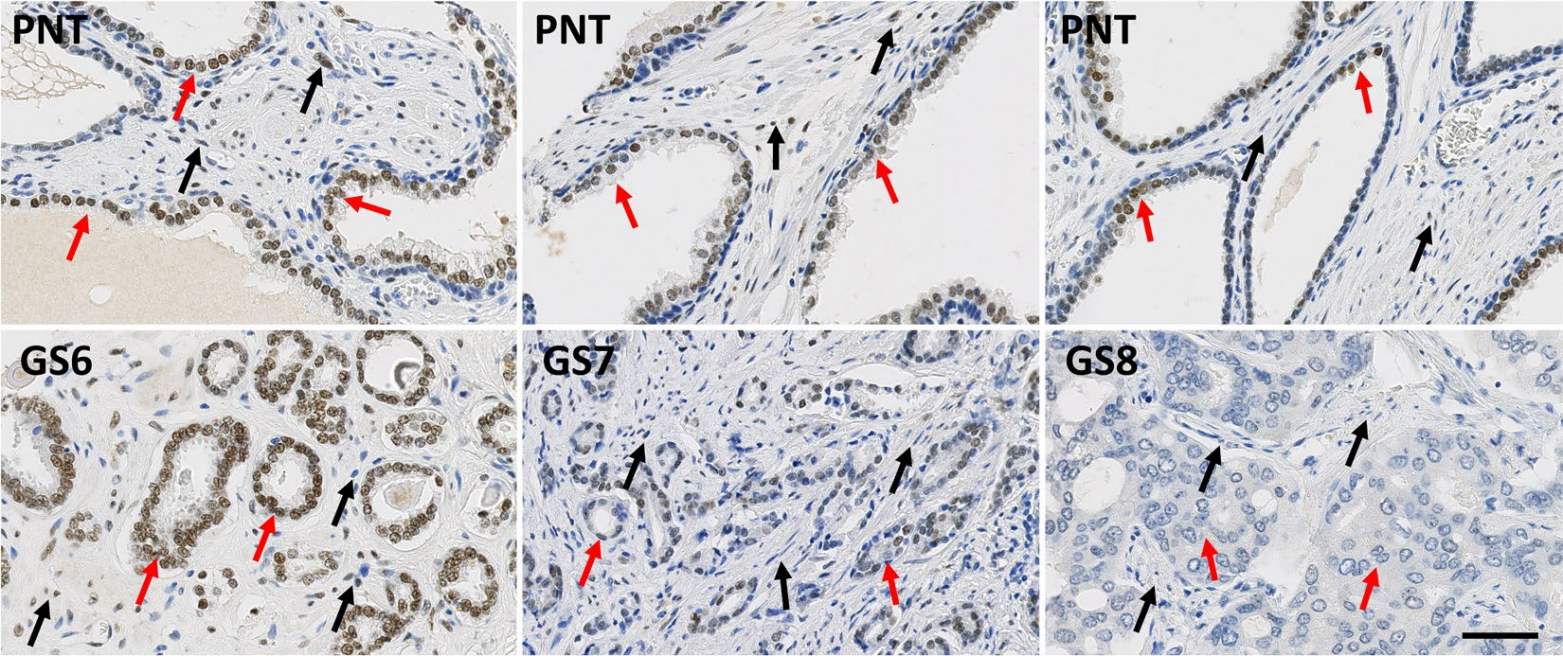
Immunohistochemical stains for AR in PCa samples (bottom) and paired PNT samples (top) from GS6-7-8 patients. AR can be expressed in epithelial (red arrows) and stromal cells (black arrows). Scale bar equals 50 µm.

**Figure 2:**
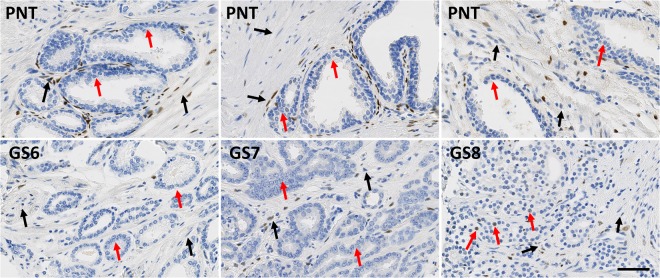
Immunohistochemical stains for ER in PCa samples (bottom) and paired PNT samples (top) from GS6-7-8 patients. ER can be expressed in epithelial (red arrows) and stromal cells (black arrows). Scale bar equals 50 µm.

**Figure 3:**
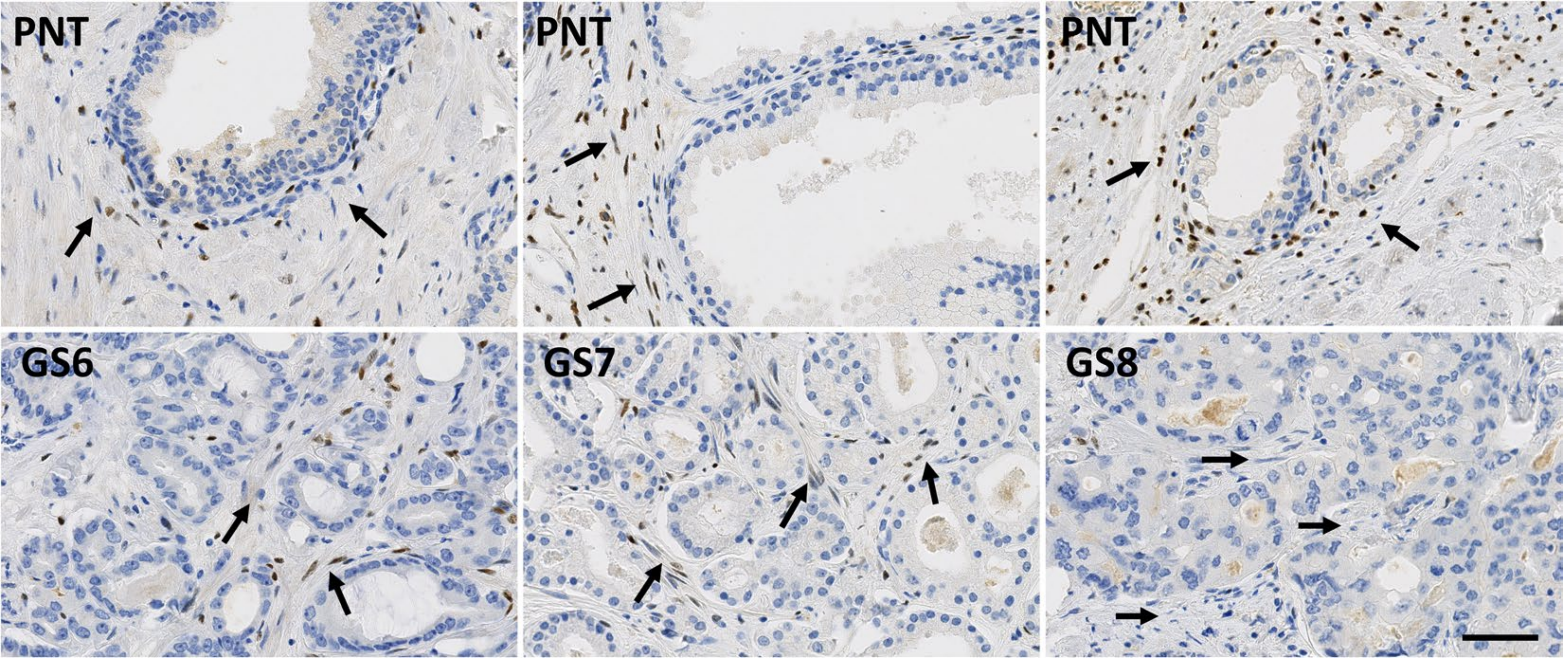
Immunohistochemical stains for PR in PCa samples (bottom) and paired PNT samples (top) from GS6-7-8 patients. PR is expressed on stromal cells (black arrows). Scale bar equals 50 µm.

**Figure 4:**
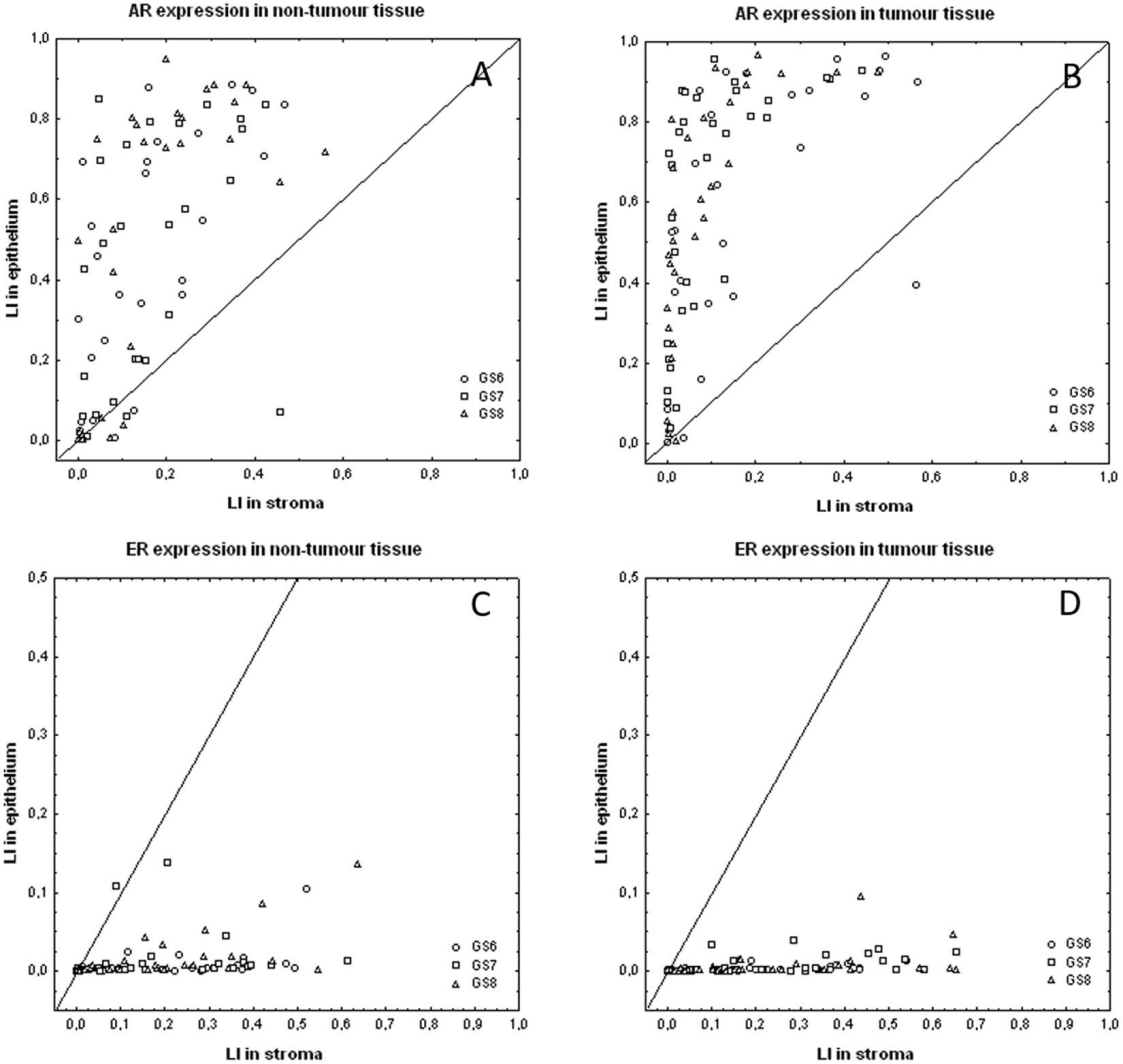
Compartmentalized expression of AR (**A**,**B**) and ER (**C**,**D**) quantified by means of the labeling index (LI) computed in stroma (X-axis) and epithelium (Y-axis) from non-tumour (**A**,**C**) and tumour tissue samples (**B**,**D**). Each symbol identifies a PCa patient classified with respect to the Gleason score of the tumour (see graph legend). The diagonal indicates equal expression in epithelium and stroma.

While PR is only expressed in stromal cells, AR and ER can be expressed in both epithelial and stromal cells (Figs 1–3). These latter two receptors exhibit differential expression between these two histological compartments in both PNT and PCa tissue, but in an opposite and contrasting way, as shown in Fig. 4. In both PNT and PCa tissue, AR expression is significantly higher in epithelium then in stroma (Sign test: p < 0.001 for both, Fig. 4A,B), whereas ER expression is significantly lower in epithelium in both PNT and PCa tissue (Sign test: p < 0.001 for both, Fig. 4C,D). Figure 4 shows that these differences are observed in a very large majority of cases (i.e. between 85% and 100%) in each GS group. For AR, the increased signal in epithelium is more drastic in the PCa than in the PNT samples, with very weak to negative expression in tumour stroma (Fig. 4A,B). Concerning ER, epithelial expression is in fact very weak in any tissue sample whereas stroma can exhibit high ER LI values (Fig. 4C,D).

**Figure 5:**
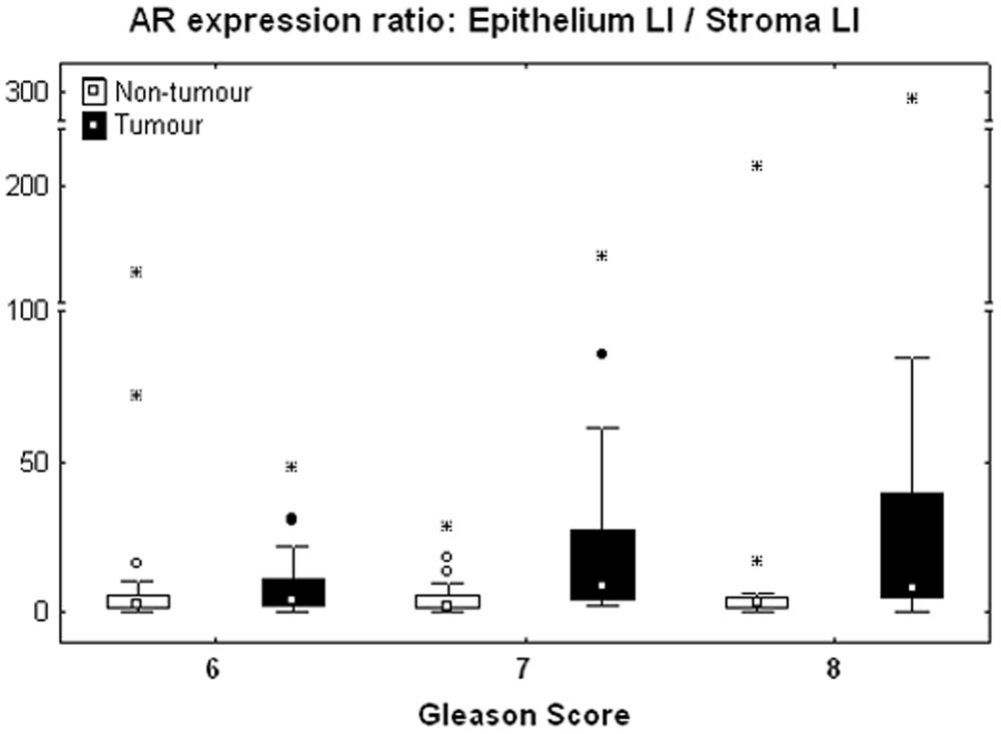
Variations of the epithelium/stroma LI ratio for AR computed per patient in the non-tumour (white boxes) and tumour (black boxes) tissue samples and shown according to the GS. The data distributions are described by means of their median (small square), interquartile range (box), non-outlier minimum and maximum values (bars) and the remaining outlier (dot) and extreme (asterisk) values.

To refine these observations, we computed the epithelium/stroma LI ratios for AR and ER and checked whether they significantly differ between PNT and PCa samples. As expected from Fig. 4, there is a significant difference for AR only, with higher ratios in tumour areas (Sign test: p < 0.001). We then analysed the GS impact on these ratios. As shown in Fig. 5, the GS significantly modifies AR LI ratios in tumour tissue only (Kruskal-Wallis test: p = 0.007), with a significant increase in GS7 (p = 0.019) and GS8 (p = 0.018) as compared to GS6 (post-hoc test). In contrast, no significant GS impact was seen for the ER LI ratios.

**Figure 6:**
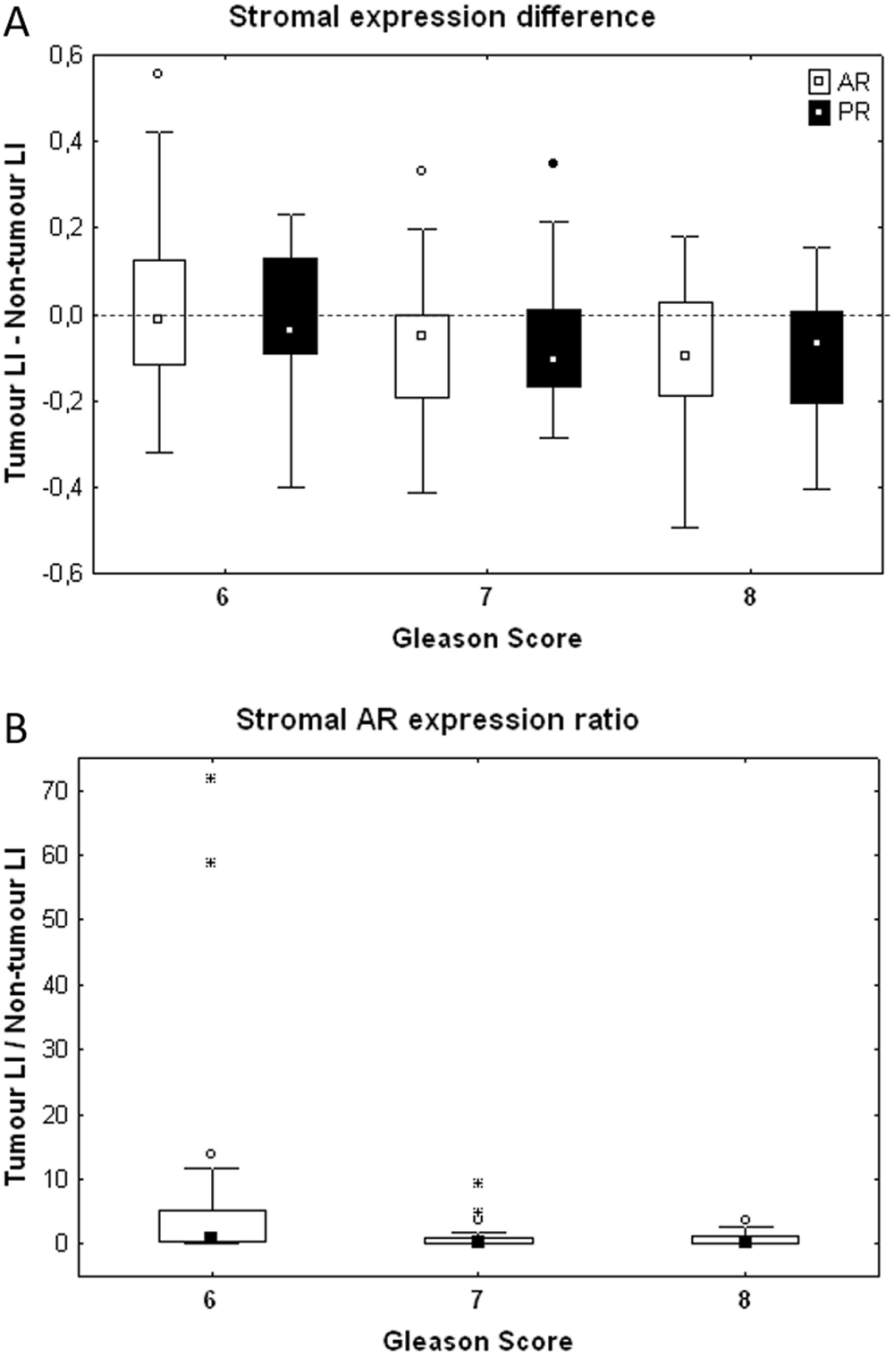
Stromal expression difference (top) and ratio (bottom) between the LI values of AR and PR measured per patient in tumour and non-tumour areas.

In PCa samples we observed no significant differences in stromal expression of AR, PR and ER nor for the epithelial expression of AR and ER across the different GS groups. However, considering the expression difference computed between the PCa and PNT samples for each patient, Fig. 6A shows similar trends for AR (white boxes) and PR (black boxes) concerning their stromal expression differences in relation to the GS. The negative values indicate that the stromal expression levels of AR and PR decrease in PCa tissue - as compared to PNT tissue - in both GS7 and GS8 but not in GS6. Because of the relatively small size of the GS groups, we performed the Sign test, which confirmed significant decreases for AR in GS7 (p = 0.007) and PR in GS8 (p = 0.031) only. In both cases, this decrease was observed for more than 70% of cases, whereas it was for a little less for each marker (between 67% and 68% of cases) in the other high GS groups. A lack of symmetry in the differences between these GS groups prevents considering the Wilcoxon test.

We refined the previous analysis by computing the tumour/non-tumour LI ratios for each (epithelial and stromal) SHR expression. The sole significant variation evidenced across the three GS groups was the tumour/non-tumour ratio of stromal AR expression, which decreases in high GSs (Kruskal-Wallis test, p = 0.032, Fig. 6B). The (more conservative) post-hoc tests only confirmed the significant decrease in GS8 as compared to GS6 (p = 0.039).

A post-analysis in which the GS7 cohort was subdivided in ISUP GG2 (GS 3 + 4) (n = 14) and ISUP GG3 (GS 4 + 3 = 7) (n = 18) did not show any significant difference in the SHR expression profiles between them.

No significant differences were found in heterogeneity of SHR expression between the GS groups. When considering all patients together (n = 90), there is a significant increase in heterogeneity of AR-expression in epithelium compared to stroma in PCa samples (observed in 62% of cases, Sign test: p = 0.034). Inversely, heterogeneity of ER-expression significantly decreases in epithelium of PCa samples (observed in 97% of cases, Sign test: p < 0.001).

**Figure 7:**
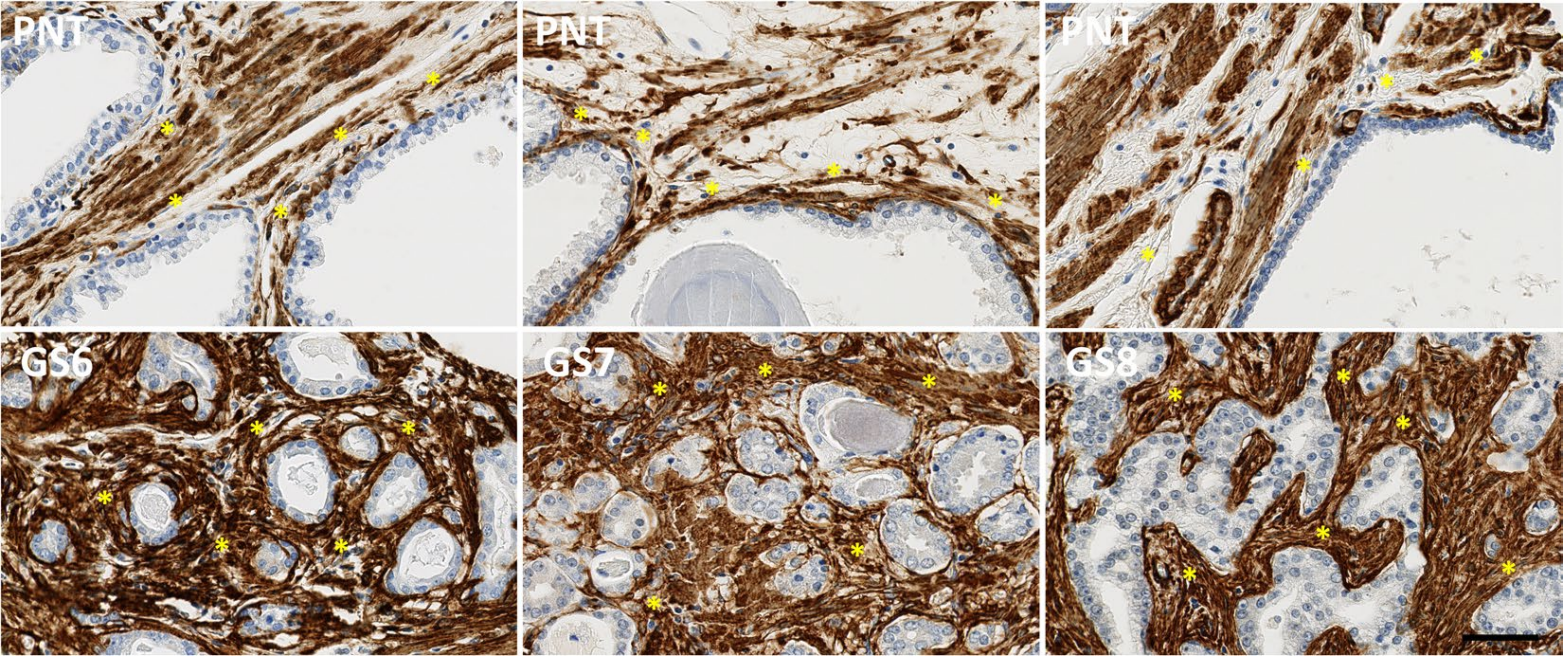
Immunohistochemical stains for αSMA in PCa samples (bottom) and paired PNT samples (top) from GS6-7-8 patients. αSMA is expressed on stromal cells (asterisks). Scale bar equals 50 µm.

**Figure 8:**
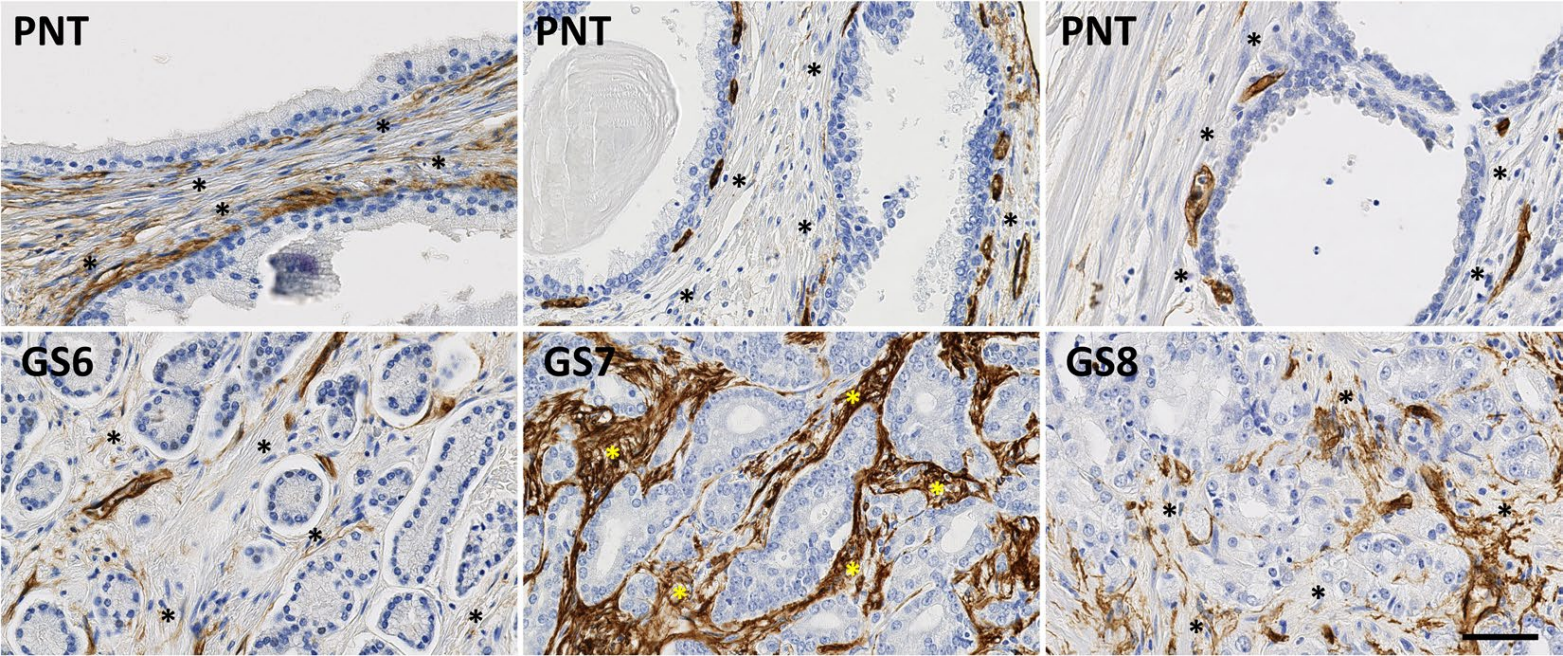
Immunohistochemical stains for CD34 in PCa samples (bottom) and paired PNT samples (top) from GS6-7-8 patients. CD34 is expressed on stromal cells (asterisks). Scale bar equals 50 µm.

**Figure 9:**
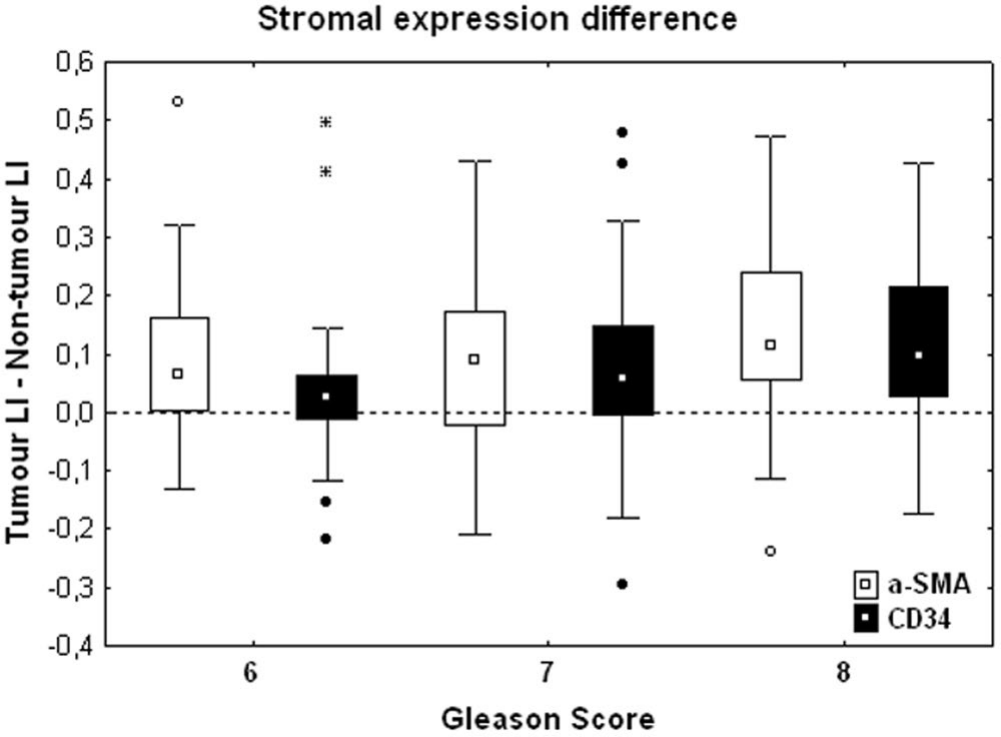
Stromal expression difference between the LI values of α-SMA (white boxes) and CD34 (black boxes) measured per patient in tumour and non-tumour areas and shown according to the GS. The rest of the legend is similar to that of Fig. 5.

The stromal expression of αSMA and CD34 (see Figs 7 and 8) significantly increases in PCa tissue - as compared to PNT tissue - in each GS group for αSMA (Sign test: p = 0.007 for GS6, p = 0.054 for GS7 and p = 0.005 for GS8) and in GS7 and GS8 only for CD34 (Sign test: p = 0.021 for GS7 and p < 0.001 for GS8) (Fig. 9). These differences significantly increase from GS6 to GS8 for CD34 only (Kruskal-Wallis: p = 0.016), in particular between GS6 and GS8 (post-hoc test: p = 0.012). This increase is in fact due to a significantly higher expression of CD34 in GS8 as compared to GS6 in the stroma of PCa tissue samples (post-hoc test: p = 0.013). In the PCa tissue samples, no significant differences were found in stromal expression of αSMA across the different GS groups.

Cav-1 expression is not significantly different between PCa and PNT tissue. No significant differences in CAF expression profiles were found between ISUP GG2 and GG3.

**Figure 10:**
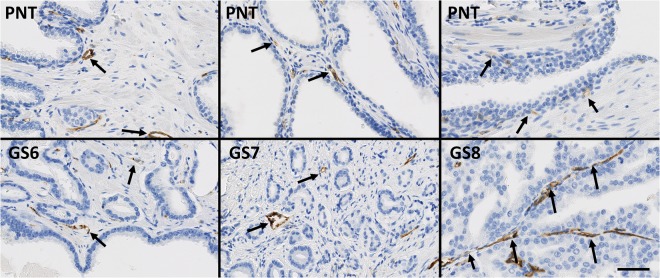
Immunohistochemical stains for CD31 in PCa samples (boffffttom) and paired PNT samples (top) from GS6-7-8 patients. CD31 is expressed in the endothelium of blood vessels (black arrows). Scale bar equals 50 µm.

**Figure 11:**
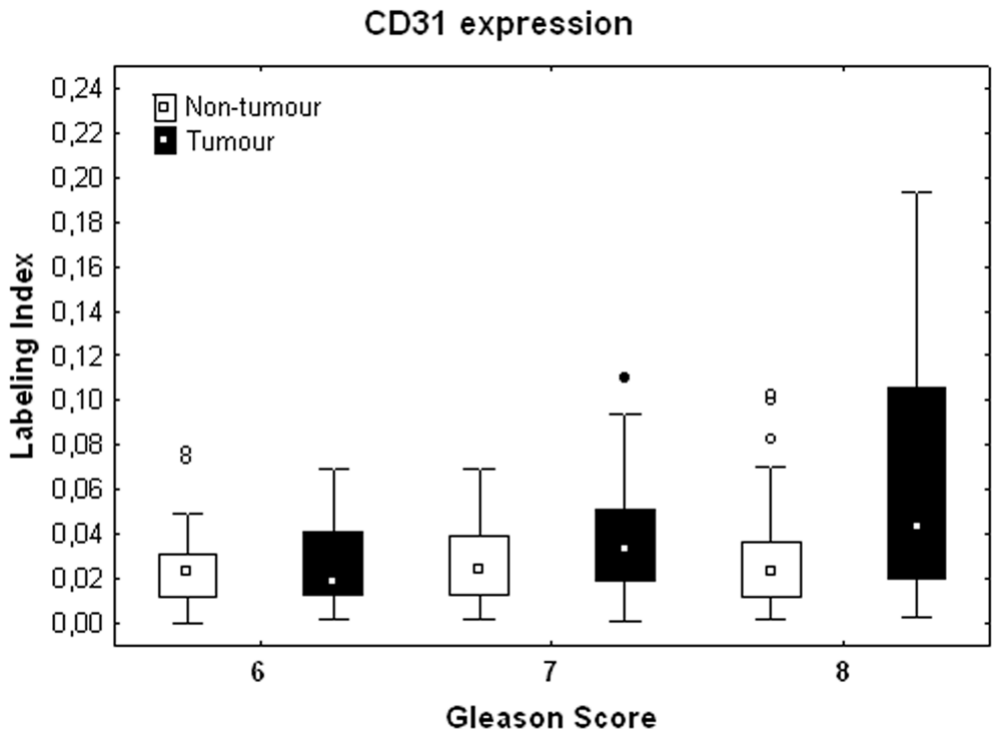
Stromal expression of CD31 in non-tumour (white boxes) and tumour (black boxes) areas and shown according to the tumor GS. The rest of the legend is similar to that of Fig. 5.

The expression of the vascular marker CD31 (see Fig. 10) significantly increases in GS8 PCa tissue compared to PNT tissue from the same patient (Sign test: p = 0.016; Fig. 11). This result is in fact related to a significantly higher expression of CD31 in GS8 compared to GS6 tumour tissue (post hoc test: p = 0.028), without variation in the PNT tissue (Fig. 11). No significant differences in CD31 expression profiles were found between ISUP GG2 and GG3.

**Figure 12:**
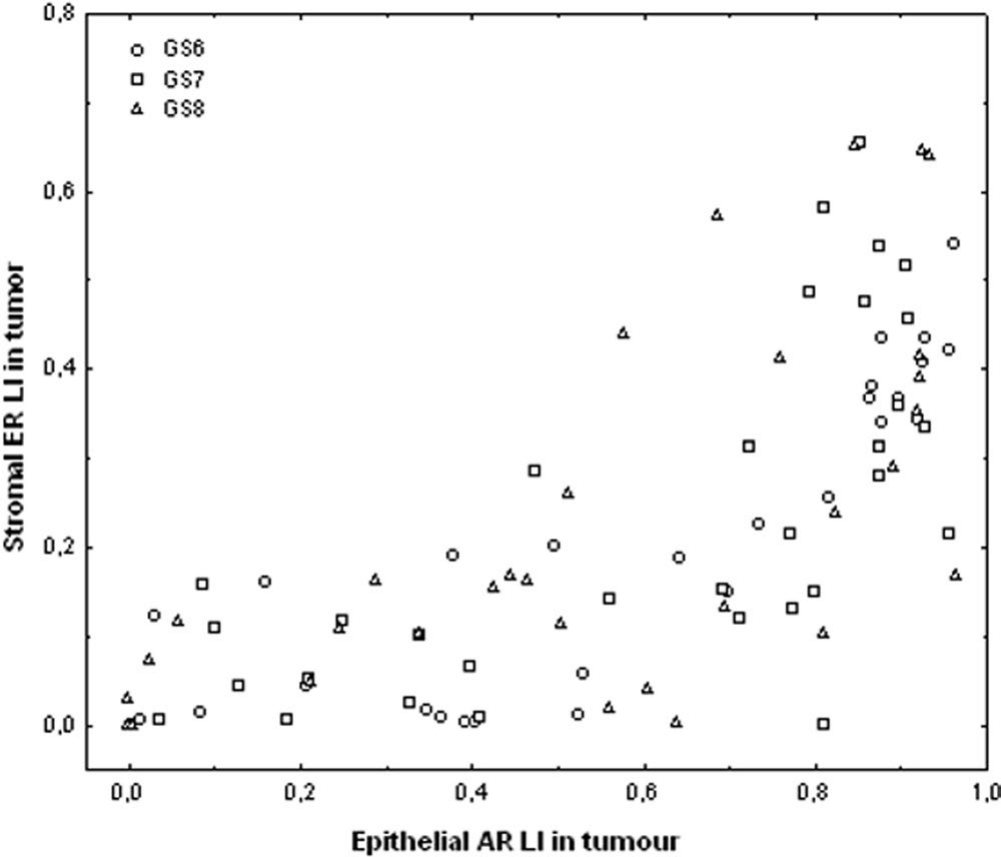
Graph showing the positive correlation between epithelial AR LI (X-axis) and stromal ER LI (Y-axis), both measured in tumour tissue areas. Each symbol identifies a PCa patient classified with respect to the GS of the tumour (see graph legend).

Since the 3 GS groups exhibit the same trends in all the Spearman correlation analyses (as illustrated in Fig. 12), they are not distinguished in the following results, which concern all the data. All the Spearman correlation indices (r_s) reported below are highly significant (p < 0.001) and focus on the strongest correlations.

Positive correlations were observed for AR between epithelial and stromal expression (for PNT and PCa samples (r_s = 0.68 and 0.75, respectively)). Similar trends were obtained for ER (r_s = 0.66 and 0.67, respectively).

Cross correlating the different SHR expression levels, positive correlations were found in tumour areas between epithelial AR and stromal ER (r_s = 0.76, see Fig. 12) and, to a lesser extent, between stromal AR and stromal PR (r_s = 0.53). Similarly, positive correlations for tumour/non-tumour LI ratios were found between epithelial AR and stromal ER (r_s = 0.60) and between stromal AR and stromal PR (r_s = 0.57).

Of the other markers, the strongest correlation results involve CD31 expression in tumour tissue, which is negatively correlated with the SHR markers in tumour, and, more particularly, the epithelial expression of AR (r_s = −0.54) and, to a lesser extent, the stromal expression of both ER (r_s = −0.41) and PR (r_s = −0.40).

We completed our correlation analysis by comparing marker expression levels and two patient features: their age and PSA level. We carried out these analyses in each GS group and found significant correlations in GS7 only; these data are summarised in Table 1

**Table 1:** Spearman correlation analysis related to patient’s age and PSA level in the GS7 group; only significant correlations are mentioned with their p-value (<0.05).

GS7 group Protein expression	AGE	PSA level
SMA in PNT	—	0.43 (0.025)
CD31 in PNT	—	0.46 (0.016)
Stromal ER in PNT	0.50 (0.009)	—
Cav-1 in PCa	−0.47 (0.007)	—

## Discussion

In this study we investigated the relation between different GS and the expression of several stromal cell markers, some of which are also expressed in epithelial cells and show interesting variations between these two cell types. Stromal cell markers were selected based on a high prevalence in PCa research, their possible clinical relevance and/or biomarker potential and the availability of specific and reliable antibodies. Thus, we applied a transversal approach, using several established stromal cell markers instead of a vertical approach, focussing on a specific signalling pathway but often without reliable/validated antibodies for clinical application.

The central aim of this study was to find distinct stromal cell profiles depending on different GS. This finding would support the existence of specific stromal changes depending on PCa grade and could potentially constitute a complementary tool to study and assess tumour differentiation. We evidenced several significant changes in expression profiles of stromal cell markers in high grade PCa (compared to low grade PCa and PNT): GS8 PCa was characterised by decreased stromal expression of AR and PR, increased epithelium/stroma ratio of AR-expression, increased expression of the CAF-marker CD34 and increased stromal vascularity. Low grade (GS6) PCa did not show any grade-dependent changes in stromal cell profile. The intermediate grade groups (GS7) showed a stromal cell profile similar to GS8, with a decreased stromal expression of AR and PR and increased expression of the CAF-marker CD34, although the changes were less significant and mainly related to PNT tissue. It is known that GS7 (4 + 3) has a much worse prognosis than GS7 (3 + 4)^1,2^ and therefore the recent ISUP grade group system subdivides GS7 in GG2 (3 + 4) and GG3 (4 + 3)^1,2^. However, we were unable to find specific stromal cell changes in GG2 compared to GG3. We found multiple strong correlations between several of the studied biomarkers, which were however always independent from the GS group. When we looked for correlations with the patients age and PSA we found some GS-dependent differences, mostly for the GS7 group, where stromal SMA expression ratio tends to decrease when PSA and/or age increase. We also carried out additional multivariate analysis based on the biomarker panel investigated in this study without obtaining additional information related to GS-related stromal cell profiles. The essential reason is that the extracted data appear as constituting a continuum across the different GS groups (as illustrated in Figs 4–6).

The present study provides new data on stromal cell changes in PCa and largely confirms previous reports, although some differences are apparent and need to be discussed. The decreased expression of AR in tumour stroma has been reported in several studies^8,9,16–18^, and some of them have also found an association between this decrease and a higher GS^9,17,18^. The decreased expression of PR in PCa tumour stroma has also been reported in previous studies^10,19^, but the association with higher GS was not found^10^, which might be due to pooling of GS6 and GS7 as one study cohort^10^. Concerning conflicting reports respectively showing a decrease^7^ and increase^20^ in stromal expression of ERα in PCa, several methodological differences, like cohort characteristics, pre-analytical tissue sample characteristics, antibody clones, scoring methodologies,… could be involved. We were unable to show any significant difference. In several studies epithelial expression of ERα was only found in PCa and HGPIN and not in PNT tissue^21,22^. We also observed very limited expression of ERα in epithelial cells of PNT samples, with a very low epithelial to stroma ratio, which has been reported in another study^20^. The observed limited and focal expression of ERα in luminal epithelial cells might have been easily overlooked by semi-quantitative assessments, as carried out in previous reports^21,22^. Despite the increasing amount of reports showing a functional role for ERβ in the onset of PCa^23^, we did not investigate the ERβ isoform, due to the lack of reliable and specific ERβ antibodies^24,25^.

Several studies have investigated the expression of Cav-1 in PCa stroma, and the majority of them has found a relation between low expression levels and poor clinical outcome^11,26^. Data on Cav-1 expression levels in PCa stroma are not uniform, with several studies reporting on decreased expression levels^12,26^, sometimes related with high GS^12^, but with other studies lacking obvious changes^11^. Apart from a negative correlation with age (Cav-1 expression decreases when age increases), we were unable to find any other significant change in Cav-1 expression levels in PCa. Possible explanations for these conflicting data are methodological differences in delineation of the stromal compartments and in scoring procedures (whereas the used antibody clones against Cav-1 were equal amongst most studies^11,12,26^). There have also been reports of epithelial overexpression of Cav-1 in high grade PCa^27,28^, but we were unable to show significant epithelial Cav-1 staining in our PCa samples, possibly due to different selection criteria for our patient samples.

The particular properties of reactive tumour stroma in PCa have been described in multiple studies^26,29,30^. An increased presence of αSMA+ and CD34+ CAF’s was repetitively reported^10,29–31^, but a relation with GS was not established yet. Earlier analysis of the relation between GS and microvessel density (MVD) resulted in conflicting reports. While some studies report a higher MVD in high grade PCa, others did not find any significant relation (for review see^32^). A critical study-parameter is the selected marker to study MVD^32^. Comparative studies have shown that CD31 is a reliable vascular marker in PCa, although CD105 (endoglin) might perform slightly better in certain conditions^33,34^. Preliminary studies in our lab with CD105 showed weak and limited vascular staining, motivating our selection of CD31 for this study.

Reliable assessment of tissue biomarkers is crucial in PCa research, but it is clear that many methodological differences between studies often hamper an adequate interpretation and comparison of the results. Amongst major issues are differences in antibody clones, staining assays and image analysis methodologies^32^. We tried to ensure as much as possible the validation and reproducibility of our staining assays, but we are well aware that in most cases no consensus exists on several methodological parameters (e.g. the choice of antibody clones) since comparative studies are mostly lacking. A major methodological novelty in this study was the use of whole slide imaging and automated image analysis tools to characterize IHC. Compared to the generally used semi-quantitative and observer-dependent visual scoring systems of IHC, annotating the regions of interest to compartmentalise the quantitative analysis is a very time-consuming approach, but it has the major advantages to generate robust and reproducible quantitative data and to avoid the effects of human subjectivity in visual evaluation^32,35–38^. Recent developments in machine learning, and more specifically in deep learning, propose methods for automatically segmenting glandular epithelium in H&E slide images from non-tumor and tumor colorectal tissue samples^39,40^. This kind of tools will greatly help to refine quantitative IHC analysis when they outperform manually compartmentalization, as done for example in the present study.

Although we cannot draw direct functional conclusions based on the present study design, the finding of specific stromal cell marker changes in high grade PCa is likely to reflect a different stromal cell behaviour depending on tumour grade and differentiation. The decreased expression levels of stromal AR and PR in high grade PCa could reflect a tumour suppressive role for these SHR. Previous studies have shown an association between low stromal AR and death from PCa, suggesting that stromal AR prevents metastasis of evolving epithelial cancer cells^8,17^. Similar findings have been reported for stromal PR^41^. The exact role of the reactive tumour stroma in modulating tumour progression is still under debate, but several studies suggest that the damage response biology of reactive stroma is likely to be tumour-promoting^42^. In this context the high amounts of CD34^+^ and SMA^+^ CAF’s in high grade PCa might reflect increased tumorigenic properties of the reactive stroma. Some studies have suggested that CD34+ stromal cells exhibit mesenchymal stem cell properties and are recruited from the microvasculature to contribute to the formation of reactive stroma in tumours like PCa^31^.

In conclusion, we have found several GS-specific alterations in stromal cell marker profiles, but we were unable to find a global GS-specific multi-marker stromal cell profile in PCa. The present data need to be confirmed in a larger and prospective series with an additional focus on multi-marker interactions. Automated image analysis tools will be crucial to obtain reproducible and robust biomarker data in future studies.

## Methods

### Patients

The study was approved by the Institution’s Ethical Committee and Biobank Board as a satellite project to the prostate enabling protocol for studies on a large retrospective prostate tissue database (S55860). The study protocol was in accordance to the EU guidelines. All patient-related sample-data were fully anonymized in the study analysis. Informed consent could not be obtained since research was performed on a retrospective tissue database. Therefore a waiver of consent was issued by the institution’s ethical committee.

Matched patient cohorts were composed of different GS (GS6 (n = 28)/ GS7 (n = 32)/ GS8 (n = 31)). Patient cohorts were matched for clinical recurrence (CR, established local and/or distant disease recurrence), lymph node-status, margin-status, p-stage and age. All cases were revised for correct GS by an experienced urogenital pathologist. Paired PNT samples were made up of tissue blocks from the same patient cohorts containing histologically normal prostate tissue (n = 91).

### Sample handling

#### Tissue micro-arrays

Tissue micro-array (TMA) layout designs were used to develop the TMAs from donor paraffin blocks. Donor paraffin blocks were collected from a large database with tissue blocks from radical prostatectomy specimens. Based on haematoxylin and eosin (H&E) stained slides, two representative paraffin blocks were selected per patient: one with PCa and one with histologically normal prostate (i.e. PNT) tissue. Per block six cylindrical cores were harvested and inserted into a recipient paraffin block with Alphelys minicore (Alphelys, France). A total amount of 6 TMAs was carried out from 91 PCa patients and consisting of paired PCa and PNT samples.

#### Immunohistochemistry (IHC)

Antibody-clones were selected for their epitope-selectivity (see Table 2 for details), most of them being extensively validated for clinical diagnostic practice (www.nordiq.com). Prior to enrolment in the study, antibodies were validated on control tissue for staining specificity and reliability. Antibodies were directed against SHR (AR, PR and ER), CAF markers (CD34, Cav-1 and αSMA) and the vascular marker CD31.

**Table 2:** Properties of the antibody clones used.

Immunogen	Clone	Manufacturer/Code	Host	Titer	Control
*Alpha-smooth muscle actin (α-sma)* (N-terminal synthetic decapeptide of α-smooth muscle actin)	1A4	Agilent Technologies, Diegem, Belgium *IR611*	Mouse	Ready to use	Appendix
*Androgen receptor (AR)* (synthetic peptide with amino acids 229–315 of the human AR)	AR441	Agilent Technologies, Diegem, Belgium *M3562*	Mouse	1/100	Prostate, Breast (non-tumour)
*Caveolin-1 (Cav-1)* (synthetic peptide at the N-terminus of human caveolin-1)	N20	Santa-Cruz Biotechnology, Heidelberg, Germany *SC-894*	Rabbit	1/100	Lung
*CD31* (cell membrane from spleen)	JC70A	Agilent Technologies, Diegem, Belgium *IR610*	Mouse	Ready to use	Appendix
*CD34* (endothelial cell membranes from human placenta)	QBend10	Agilent Technologies, Diegem, Belgium*IR632*	Mouse	Ready to use	Appendix
*Estrogen receptor alpha (ER)* Soluble recombinant human estrogen receptor	1D5	Agilent Technologies, Diegem, Belgium *IS657*	Mouse	Ready to use	Uterine cervix
*Progesterone receptor (PR)* Full length A-form of human progesterone receptor	PGR636	Agilent Technologies, Diegem, Belgium M3569	Mouse	Ready to use	Uterine cervix

TMAs were cut in serial slides of 5μm to have all markers evidenced on similar tissue areas. IHC stains were done with the automated Leica Bond-Max system (Leica Microsystems, Belgium). The automated procedure consisted of: blocking endogenous peroxidase activity using 0.3% H_2_O_2_ in methanol, heat-induced antigen retrieval, incubation with primary antibodies for 15 min, incubation with a peroxidase-labelled polymer during 30 min and a subsequent incubation with a substrate-chromogen (mixed DAB refine) for 10 min. Nuclear counterstaining was done with haematoxylin.

#### Compartmentalized and quantitative staining analysis

To avoid bleaching, within 2 weeks after staining, the TMA slides were digitized at 20x using a calibrated whole slide scanner (NanoZoomer 2.0-HT, Hamamatsu, Hamamatsu City, Japan). The calibration concerns light intensity, white balance and shading and is done every day automatically, using a special slide provided by the manufacturer. Annotations were then performed by an experienced urogenital pathologist using the Visiopharm software package (Visiopharm, Hoersholm, Denmark) following stringent parameters, as detailed below.

Since some of the evaluated markers (AR and ER) can be expressed by both epithelial and stromal cells, we used different types of annotations to distinguish epithelial and stromal expression (see Supplementary Fig. 1). For the epithelial markers, normal and tumour prostate glands were selectively annotated in the PNT and PCa sample cohorts respectively. Areas with non-specific intraluminal staining were excluded from the annotated areas. For the stromal markers, the stroma contiguous with the normal and tumour prostate glands was selectively annotated. Based on previous work on the reactive stroma in PCa^29,30^, annotations were restricted to the 2–3 stromal cell layers adherent to the prostate glands (see Supplementary Fig. 1). Areas with inflammatory cells were excluded from the annotated regions. Several markers were also expressed on blood vessel components (CD34, αSMA, Cav-1), which were therefore excluded from the stromal areas submitted to analysis. It should be noted that the annotations were made on serial virtual slides in order to select similar areas from one marker to another.

A quantitative staining analysis was then performed within the annotated areas using the Visiomorph software package (Visiopharm, Hoersholm, Denmark). For each IHC marker we computed the labeling index (LI). These measurements were computed per patient, as detailed previously^43^. For the IHC markers with cytoplasmic expression (CD31, CD34, αSMA, Cav-1), the LI is the percentage of the immunostained (i.e. positive) tissue area, whereas for the IHC markers with nuclear expression (AR, PR, ER), LI is computed on the nuclear area only^43^. High LI values (expressed in percentages) are indicative of high percentages of positive cells. In view of the different tissue types considered for each marker (PCa vs. PNT and also, for some markers, epithelial vs. stromal), differences and ratios of quantitative expression (LI) values were also computed to refine the analysis of the expression variations between the different tissue components. While the differences, which are at the basis of the sign test, evidence the directions of the variations between two components, the ratios enable to take into account the proportional increase or decrease with respect to a basal expression level, e.g. in PNT tissue.

We also characterised marker expression heterogeneity in each histological compartment for each patient. To this aim we computed the LI per TMA core and their value range (=max - min) observed across the available cores per patient. We used the value range instead of the standard deviation because of the low number of cores per patient.

#### Statistics

All of the statistical analyses were performed using Statistica software (StatSoft, Tulsa, OK, USA). Paired and independent groups of quantitative data were compared using non-parametric tests, i.e. the sign test and the Kruskal-Wallis test (this latter with associated post-hoc tests), respectively. To take into account the difference extends in paired group analysis, we also checked whether the difference symmetry condition required for applying Wilcoxon matched pair test was satisfied. Correlation between staining features was investigated using Spearman correlation analysis.

## Electronic supplementary material

Supplementary Figure 1
