# Supplementary figures and images for: Comparing the expression profiles of steroid hormone receptors and stromal cell markers in prostate cancer at different Gleason scores
Source: Sci Rep. 2018 Sep 25;8:14326. doi: 10.1038/s41598-018-32711-9 (PMC6156570; doi:10.1038/s41598-018-32711-9)

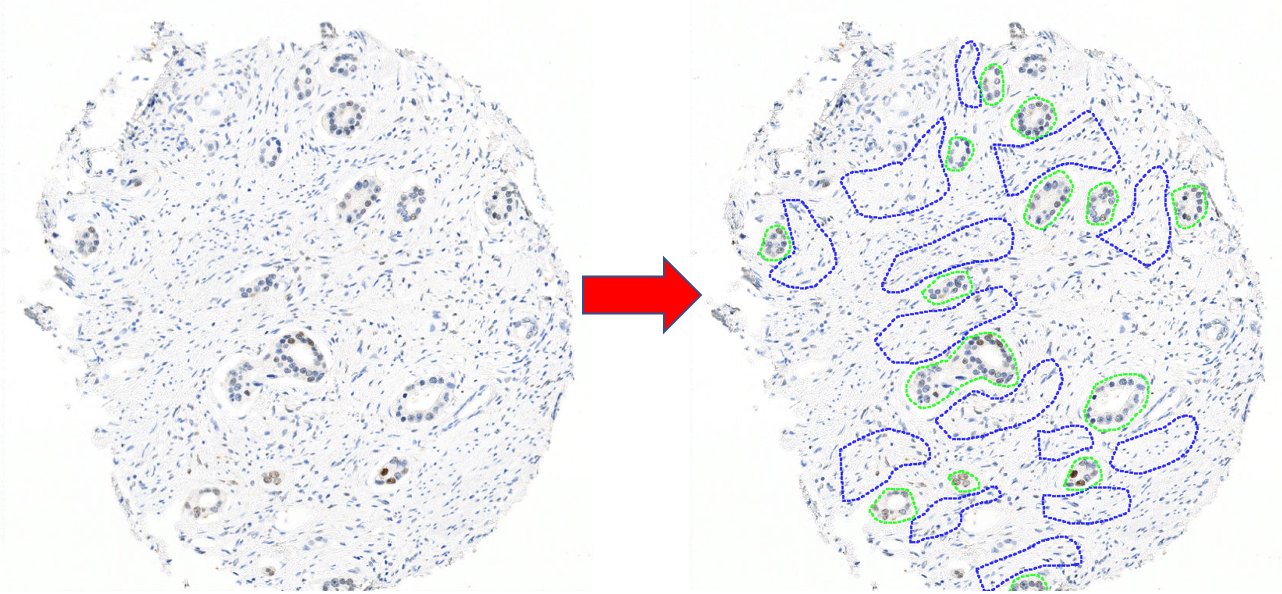

Supplement: Supplementary file 1 — Supplementary Figure 1 [file 41598_2018_32711_MOESM1_ESM.tif]
